# Context-Dependent Alterations of E-Cadherin, β-Catenin, and Vimentin in Endometrial Ciliated Epithelial Change: An Exploratory Immunohistochemical Study

**DOI:** 10.3390/diagnostics16111646

**Published:** 2026-05-27

**Authors:** Teona Turashvili, George Tevdorashvili, George Burkadze

**Affiliations:** Department of Pathology, Tbilisi State Medical University, Tbilisi 0186, Georgia

**Keywords:** ciliated epithelial change, endometrium, E-cadherin, β-catenin, vimentin, immunohistochemistry, digital pathology, tubal metaplasia, FOXJ1, multiciliogenesis, molecular classification

## Abstract

**Background/Objectives:** Ciliated epithelial change in endometrial lesions is a recognized morphologic finding, but its immunophenotypic correlates and biological significance remain insufficiently defined. We investigated whether endometrial lesions with ciliated epithelial change show reproducible immunohistochemical alterations across benign, premalignant, and malignant diagnostic categories. **Methods:** We performed a retrospective immunohistochemical study of 315 formalin-fixed paraffin-embedded eutopic uterine endometrial specimens (no endometriotic/ectopic lesions included) collected between 2019 and 2024 and distributed equally across seven diagnostic categories (*n* = 45 each): normal endometrium, endometrial polyp, hyperplasia with cystic/disordered glands, hyperplasia with crowded glands, atypical hyperplasia/EIN, endometrioid carcinoma, and serous carcinoma. Marker expression was quantified by digital image analysis and compared between lesions with and without ciliated epithelial change, including lesions with ciliated epithelial change showing cytological atypia. **Results:** Ciliated epithelial change (CEC) was identified in 86/315 cases (27.3%), including 41 cases (13.0%) with atypical CEC. In benign categories, lesions with CEC showed lower E-cadherin expression and higher β-catenin expression, including more frequent nuclear β-catenin localization. In carcinomas, these patterns were not recapitulated and instead showed an opposite or attenuated profile, supporting a context-dependent rather than linear model. Vimentin was consistently reduced in lesions with CEC across diagnostic categories. p53 and CD44 showed heterogeneous findings and were less informative than the adhesion- and phenotype-related markers. **Conclusions:** Endometrial lesions with CEC show reproducible, context-dependent immunohistochemical alterations, most consistently involving E-cadherin, β-catenin, and vimentin. In particular, nuclear β-catenin reactivity in this setting should not be interpreted as evidence of canonical Wnt-pathway activation in the absence of CTNNB1 sequencing or validated downstream readouts, and the carcinoma findings cannot be assigned to a specific TCGA/ProMisE molecular subgroup using immunohistochemistry alone. The observations should therefore be regarded as exploratory and warrant validation in studies incorporating molecular classification, direct ciliogenesis markers (FOXJ1, acetylated α-tubulin, basal body markers), and outcome data.

## 1. Introduction

Endometrial cancer represents a major global health burden, ranking as the sixth most common malignancy in women worldwide, with incidence and mortality rates increasing in recent decades [1]. Understanding early pathological changes that precede or accompany malignant transformation is critical for developing improved risk stratification strategies.

Ciliated epithelial change in the endometrium is characterized by glands lined, in whole or in part, by columnar cells with apical cilia and has been described in normal cycling endometrium as well as in polyps, hyperplasia, and carcinoma [2,3]. The terminology requires careful handling in gynecologic pathology, because ciliated cells are a physiologic constituent of the endometrial epithelium, particularly in hormonally responsive settings. Accordingly, some examples likely represent accentuation of a native differentiation program rather than true metaplasia in the strict biologic sense. We nevertheless retain the conventional term “ciliary metaplasia” where relevant because it remains embedded in diagnostic usage and in the WHO classification, while using the more descriptive phrase “ciliated epithelial change” (CEC) throughout the manuscript when discussing the study findings [4]. Recent single-cell transcriptomic atlases of the human endometrium have established that ciliated cells are an intrinsic, hormonally regulated component of the eutopic endometrial epithelium that expands during the proliferative phase under estrogenic drive and contracts as progesterone dominance emerges in the secretory phase [5,6,7]. CEC therefore encompasses a continuum that ranges from physiologic, estrogen-driven multiciliation through tubal-type metaplasia (true reprogramming toward fallopian-tube-like differentiation) to the rare ciliated-cell variant of endometrioid carcinoma [8,9,10,11]. Whether such change, particularly when accompanied by cytologic atypia, is associated with reproducible molecular or immunophenotypic alterations remains insufficiently defined.

Because β-catenin can shuttle from the membrane to the nucleus in response to Wnt-axis activation, hormonal signaling, junctional remodeling and post-translational events [12,13], we anticipated that nuclear β-catenin reactivity, while suggestive of Wnt activity, would not be specific for it; we have therefore interpreted such patterns cautiously throughout. We further note that endometrial carcinoma is now classified within the integrated TCGA/ProMisE molecular framework into POLE-ultramutated, mismatch-repair-deficient, p53-abnormal, and no specific molecular profile (NSMP) subgroups [14,15,16,17,18], and that CTNNB1 mutations cluster within the NSMP subgroup [18,19,20]. Although the present immunohistochemical study cannot resolve molecular subgrouping, this framework anchors the interpretation of our carcinoma findings.

Despite the potential biological interest of CEC, systematic immunohistochemical studies across the spectrum of endometrial pathology remain limited. We therefore examined a broad marker panel in a retrospective cohort spanning benign, premalignant, and malignant lesions, with the aim of determining whether CEC is associated with reproducible alterations in adhesion-related, phenotype-related, proliferation-related, and hormone receptor markers. Given the absence of parallel sequencing or full molecular classification, this study was designed as an exploratory pathology investigation intended to generate testable biologic hypotheses rather than to establish mechanism or prognostic value.

## 2. Materials and Methods

### 2.1. Study Design and Ethical Approval

This retrospective cohort study utilized archival tissue from the teaching-scientific and diagnostic laboratory at Tbilisi State Medical University, Tbilisi, Georgia, spanning 2019–2024. All material analyzed comprises eutopic uterine endometrial specimens (hysterectomy or curettage). The cohort does not include any endometriotic (ectopic) lesions, whether ovarian, peritoneal, or deep-infiltrating, nor any other non-eutopic or non-uterine endometrial-type tissue. The study therefore evaluates ciliated epithelial change exclusively within in situ endometrium spanning benign (non-neoplastic), premalignant, and malignant categories as listed in Section 2.3, and findings should not be extrapolated to endometriotic lesions, which represent a biologically distinct ectopic endometrial process. The study protocol received approval from the Institutional Review Board of Tbilisi State Medical University (Approval Number: #480942; date of approval: 24 December 2020). Due to the retrospective nature of the study and the use of de-identified archival material, the requirement for informed consent was waived by the ethics committee in accordance with institutional guidelines and the Declaration of Helsinki.

### 2.2. Sample Size Justification

Sample size was determined based on prior immunohistochemical studies of E-cadherin and β-catenin in endometrial pathology, which demonstrated effect sizes (Cohen’s d) of 0.8–1.2 for comparisons between diagnostic categories [21]. With α = 0.05 and power = 0.80, a minimum of 26 cases per group was required to detect large effect sizes. We included 45 cases per diagnostic category to ensure adequate power for subgroup analyses involving comparisons between cases with and without CEC.

### 2.3. Sample Selection and Classification

The study included 315 formalin-fixed paraffin-embedded endometrial tissue specimens distributed equally (*n* = 45) across seven diagnostic categories: normal endometrium (stratified by menstrual cycle phase where determinable), endometrial polyps, hyperplasia with cystic/disordered glands (hyperplasia without atypia), hyperplasia with crowded glands (hyperplasia without atypia), atypical hyperplasia/EIN, endometrioid carcinoma, and serous carcinoma.

Inclusion criteria were as follows: (1) sufficient formalin-fixed paraffin-embedded tissue for preparation of 10–12 serial sections; (2) well-preserved tissue architecture without significant processing artifacts; and (3) unambiguous diagnostic classification confirmed by institutional consensus review. Cases with mixed histological subtypes (e.g., mixed endometrioid–serous carcinoma) were excluded. The first 45 consecutive cases meeting criteria in each diagnostic category were selected to minimize selection bias. All cases were classified according to the 2020 WHO Classification of Female Genital Tumours [4]. For endometrioid carcinoma cases, tumor grade was recorded using FIGO grading; all serous carcinomas were considered high-grade by definition. The distribution of endometrioid carcinoma grades was as follows: Grade 1 (*n* = 18, 40%), Grade 2 (*n* = 16, 35.6%), and Grade 3 (*n* = 11, 24.4%).

Throughout this manuscript, the phrase “carcinoma with ciliated epithelial change” denotes carcinomas containing morphologically recognizable ciliated foci. This wording is descriptive and should not be interpreted as implying a separate WHO-recognized histologic subtype. The study addresses immunophenotypic associations within tumors showing ciliated differentiation, not a new diagnostic entity.

### 2.4. Patient Demographics

Patient ages ranged from 35 to 68 years (mean ± SD: 51.7 ± 10.3 years). The distribution by menopausal status included 89 premenopausal women (28.3%), 74 perimenopausal women (23.5%), and 152 postmenopausal women (48.3%). ‘Perimenopausal’ is defined here according to the Stages of Reproductive Aging Workshop + 10 (STRAW + 10) consensus criteria [22], denoting Stages −2 to +1a, that is, the period beginning with persistent menstrual cycle-length variation of ≥7 days between consecutive cycles and extending to 12 months after the final menstrual period.

### 2.5. Diagnostic Criteria for Ciliated Epithelial Change

Ciliated epithelial change was identified on hematoxylin-eosin-stained sections as the presence of ciliated columnar epithelial cells within endometrial glands, characterized by distinct apical cilia visible at ×400 magnification. Atypical ciliated epithelial change was defined by the presence of cilia in conjunction with cytological atypia including nuclear enlargement (>1.5× adjacent non-ciliated cells), hyperchromasia, or irregular nuclear contours. All cases were reviewed independently by two board-certified pathologists (G.B. and G.T.) with subspecialty training in gynecologic pathology, each blinded to the immunohistochemistry results during CEC scoring. Inter-observer agreement was assessed using Cohen’s kappa statistic. Discordant cases (*n* = 23, 7.3%) were reviewed jointly at a multiheaded microscope to achieve consensus.

To minimize selection bias, region-of-interest (ROI) selection for digital quantification was performed by a third investigator who was blinded to CEC status. The median number of ROIs analyzed per case was 6 (interquartile range 4–8), with each ROI sized at approximately 0.16 mm^2^ and selected to represent the dominant glandular morphology. In cases with CEC, paired ROIs were annotated within the same section to capture areas with and without ciliated change at matched magnification. Cases with fewer than three evaluable ROIs were excluded from quantitative analysis.

### 2.6. Immunohistochemical Staining

Serial 4 μm tissue sections were deparaffinized in xylene and rehydrated through graded alcohols. Heat-induced antigen retrieval was performed in 10 mM citrate buffer (pH 6.0) at 95 °C for 20 min. Immunohistochemistry was performed using an automated platform (Bond-Max, Leica Biosystems, Wetzlar, Germany) with commercially available primary antibodies. The antibody panel comprised E-cadherin (clone NCH-38, 1:100, Dako, Glostrup, Denmark), β-catenin (clone β-catenin-1, 1:200, Dako), p53 (clone DO-7, 1:100, Dako), CD44 (clone DF1485, 1:100, Dako), vimentin (clone V9, 1:200, Dako), estrogen receptor (clone 1D5, 1:100, Dako), progesterone receptor (clone PgR636, 1:100, Dako), Ki-67 (clone MIB-1, 1:100, Dako), BCL2 (clone 124, 1:100, Dako), and cyclin D1 (clone SP4, 1:100, Thermo Fisher Scientific, Waltham, MA, USA); the corresponding catalog numbers and antigen-retrieval conditions are provided in Supplementary Table S2. Primary antibodies were visualized using a polymer-based detection system with 3,3′-diaminobenzidine chromogen. Sections were counterstained with hematoxylin. All staining was performed in batches with positive and negative controls included in each run to monitor inter-batch variability.

Positive controls included endometrial carcinoma tissue (for all markers), placental tissue (E-cadherin, β-catenin), and tonsil (Ki-67, p53). Negative controls were performed by omitting the primary antibody.

### 2.7. Mismatch Repair Protein Assessment in Carcinomas

In all carcinoma cases (endometrioid and serous, *n* = 90), mismatch repair (MMR) protein status was assessed by immunohistochemistry using antibodies against MLH1 (clone ES05, 1:50, Dako), PMS2 (clone EP51, 1:40, Dako), MSH2 (clone FE11, 1:100, Dako), and MSH6 (clone EP49, 1:100, Dako). Loss of expression was defined as absence of nuclear staining in tumor cells with intact internal control (stromal cells and/or lymphocytes). MMR-deficient (dMMR) status was defined as loss of at least one MMR protein.

### 2.8. Immunohistochemical Scoring

For each marker, the percentage of positive cells was calculated as (positive cells/total cells) × 100. Positivity was defined as any chromogen staining intensity above background level. For E-cadherin and β-catenin, subcellular localization was specifically recorded: membranous staining, cytoplasmic staining, and nuclear staining. Nuclear β-catenin positivity was defined as distinct nuclear staining in ≥5% of epithelial cells [23]. As emphasized in the Introduction and explored in Section 4.3, nuclear β-catenin reactivity in this setting should not be interpreted as direct evidence of canonical Wnt-pathway activation in the absence of CTNNB1 sequencing or validated downstream Wnt target read-outs [12,13,24]. For p53, immunoreactivity was classified using established patterns: (1) wild-type pattern: heterogeneous nuclear positivity in variable proportions of cells; (2) mutant-type overexpression: strong diffuse nuclear positivity in >80% of cells; or (3) null pattern: complete absence of nuclear staining with confirmed positive internal control (stromal cells) [25]. Given the known potential for enhanced wild-type p53 staining in metaplastic and reactive epithelium [26,27], particular care was taken to distinguish true aberrant overexpression from enhanced wild-type pattern. Cases with equivocal p53 patterns were reviewed by all three pathologists to reach consensus classification.

### 2.9. Digital Image Analysis

Whole slide images were acquired using a digital slide scanner (Aperio AT2, Leica Biosystems, wetzlar, germany) at ×40 magnification (0.25 μm/pixel resolution). Automated cell detection and classification was performed using QuPath software (version 0.4.3) [28]. Cell detection parameters were optimized for endometrial tissue through iterative testing on a training set of 15 cases (not included in the analysis cohort). Optical density thresholds for positivity were determined by receiver operating characteristic (ROC) analysis comparing automated versus manual scoring by two pathologists (inter-observer κ = 0.84). The correlation between QuPath automated quantification and manual counting was r = 0.92 (*p* < 0.001) in the validation cohort. A minimum of 1000 cells were counted per ROI where tissue adequacy permitted. Areas with artifacts, tissue folding, or poor preservation were excluded from analysis.

### 2.10. Statistical Analysis

Statistical analyses were performed using SPSS Statistics version 29.0 (IBM Corp., Armonk, NY, USA) and R version 4.3.0 (R Foundation for Statistical Computing, Vienna, Austria). Normality was assessed using the Shapiro–Wilk test; outliers were identified using the interquartile range method (>1.5 × IQR) and verified by visual inspection of box plots. Continuous variables are expressed as mean ± SD or median (interquartile range) as appropriate. Comparisons between groups with and without CEC within each diagnostic category were performed using independent samples t-tests (normally distributed data) or Mann–Whitney U tests (non-parametric data). Effect sizes were calculated as Cohen’s d for continuous outcomes.

Given the exploratory nature of this study with multiple comparisons across diagnostic categories and markers, the Benjamini–Hochberg procedure was applied to control the false discovery rate (FDR) at 0.05 [29]. A total of 140 primary comparisons were performed (10 markers × 7 diagnostic categories × 2 metaplasia status comparisons). Adjusted *p*-values (q-values) are reported throughout. Fold changes were calculated as ratios between expression values in lesions with and without CEC within each diagnostic category. Results with adjusted *p* < 0.05 were considered statistically significant. Given the hypothesis-generating nature of this study, all findings require validation in independent cohorts with molecular confirmation.

## 3. Results

### 3.1. Sample Characteristics and CEC Prevalence

The study cohort comprised 315 endometrial specimens distributed equally across seven diagnostic categories (*n* = 45 each). Inter-observer agreement for diagnosis of CEC was excellent (κ = 0.87, 95% CI: 0.81–0.93). CEC was identified in 86 cases (27.3%), while atypical CEC was present in 41 cases (13.0%). The frequency of CEC by diagnostic category was as follows: normal endometrium, 8/45 (17.8%); endometrial polyps, 15/45 (33.3%); hyperplasia with cystic/disordered glands, 17/45 (37.8%); hyperplasia with crowded glands, 14/45 (31.1%); atypical hyperplasia/EIN, 12/45 (26.7%); endometrioid carcinoma, 11/45 (24.4%); and serous carcinoma, 9/45 (20.0%). Patient characteristics by diagnostic category are summarized in Table 1.

### 3.2. Mismatch Repair Status in Carcinomas

Among the 90 carcinoma cases, MMR status assessment revealed 23 cases (25.6%) with MMR deficiency (dMMR). The distribution differed by histotype: 19/45 (42.2%) endometrioid carcinomas were dMMR compared with 4/45 (8.9%) serous carcinomas (*p* < 0.001). Among dMMR endometrioid carcinomas, the most common pattern was combined MLH1/PMS2 loss (*n* = 14, 73.7%), consistent with likely MLH1 promoter hypermethylation, followed by MSH2/MSH6 loss (*n* = 4, 21.1%) and isolated PMS2 loss (*n* = 1, 5.3%). Detailed MMR status by CEC status and histotype is provided in Table 2. No significant association was observed between MMR status and the presence of CEC within either histological subtype.

### 3.3. E-Cadherin Expression

**Figure 1:**
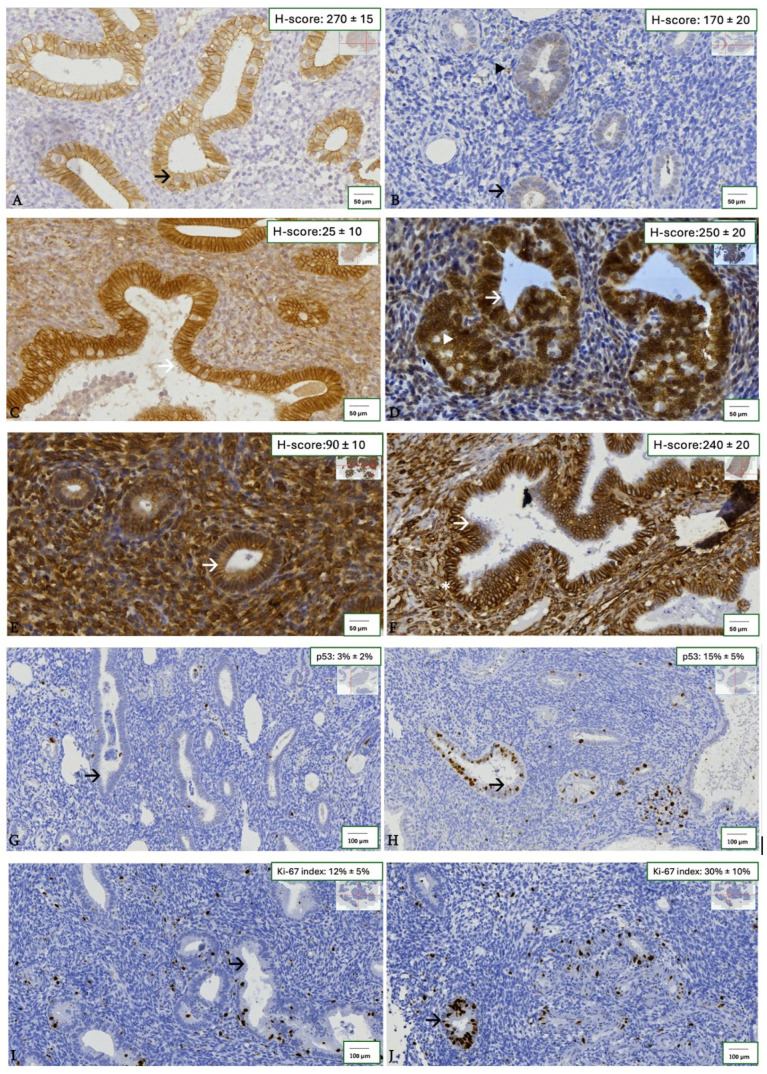

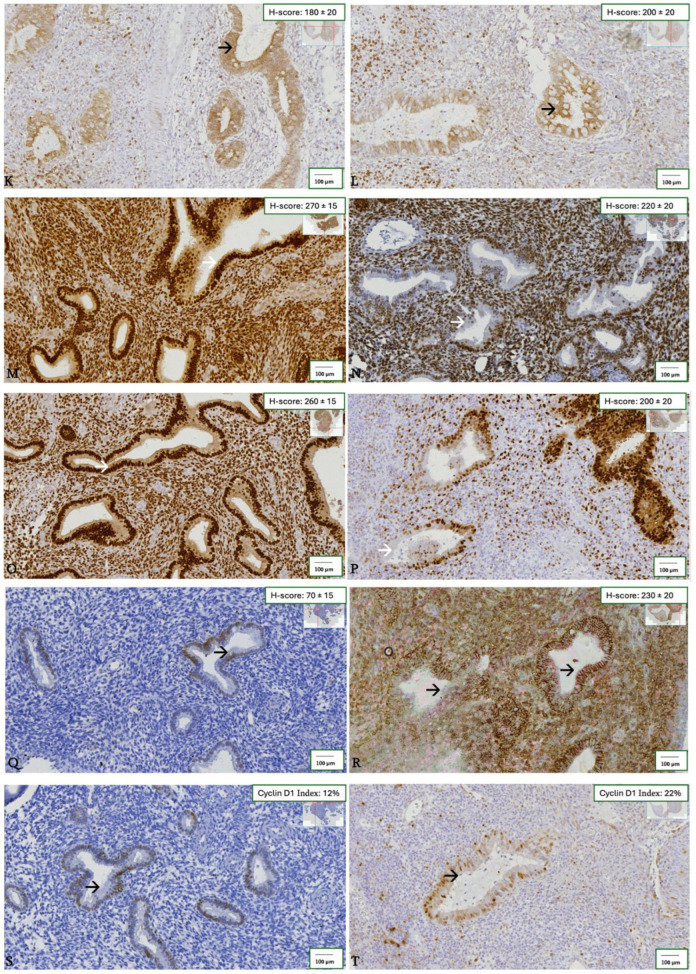
Representative photomicrographs of immunohistochemical expression in CEC and CEC with cytological atypia, expanded to include all markers in paired (CEC vs. non-CEC) format. Panels (**A**,**B**) E-cadherin (membranous, with reduction in CEC indicated by arrowheads). (**C**,**D**) β-catenin (replaced with higher-resolution images at ×400 with insets unambiguously distinguishing membranous from nuclear localization; open arrows mark nuclear β-catenin). (**E**,**F**) vimentin (asterisks mark loss-of-vimentin foci within otherwise vimentin-positive epithelium; stromal cells serve as internal positive control). (**G**,**H**) p53. (**I**,**J**) Ki-67. (**K**,**L**) BCL2. (**M**,**N**) ER. (**O**,**P**) PR. (**Q**,**R**) CD44. (**S**,**T**) cyclin D1. Solid arrows (→), ciliated cells; arrowheads (⇒), reduction in membranous E-cadherin expression in CEC relative to adjacent non-ciliated asterisks (*), foci of vimentin loss within otherwise vimentin-positive epithelium, with stromal cells serving as internal positive control; circles (o) All images at ×200 unless otherwise stated; scale bars 100 μm. Quantitative H-score insets (mean ± SD across 5 ROIs per case) are shown for each marker. Original magnification ×400 for high-power inserts.

E-cadherin expression analysis revealed consistent reductions in lesions harboring CEC within benign categories (Table 3). In endometrial polyps with CEC, membranous E-cadherin expression decreased by 21.0% compared with polyps without CEC (mean: 62.3% vs. 78.9%, 95% CI for difference: −22.4 to −10.8%; adjusted *p* = 0.003; Cohen’s d = 0.89). More pronounced reduction of 25.3% was observed in polyps with atypical CEC (mean: 58.9% vs. 78.9%, 95% CI: −26.1 to −13.9%; adjusted *p* = 0.001; d = 1.12). Representative immunohistochemical patterns are shown in Figure 1.

### 3.4. β-Catenin Expression and Subcellular Localization

**Figure 2:**
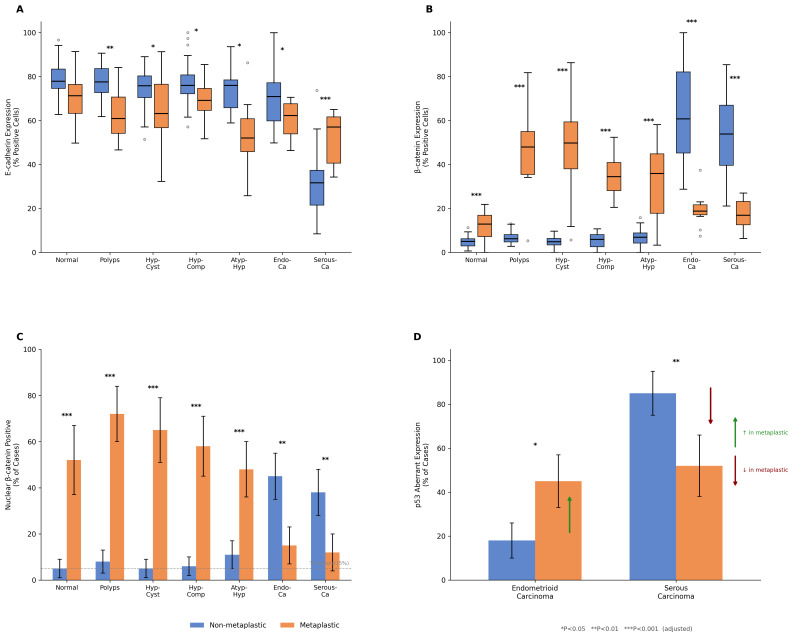
Comparative expression patterns of key markers across seven diagnostic categories. (**A**) Box plots showing E-cadherin expression (% positive cells) in lesions with versus without CEC across diagnostic categories. Note the consistent reduction in benign categories and the reversed pattern in serous carcinoma. (**B**) β-catenin expression demonstrating marked elevation in benign lesions with CEC and attenuation or reversal in carcinomas. (**C**) Proportion of cases with nuclear β-catenin localization, highlighting high rates in benign lesions with CEC and lower rates in carcinomas with CEC. (**D**) p53 expression patterns in carcinomas are shown for exploratory comparison only and should be interpreted with caution after blinded pattern-based re-review. Error bars represent 95% confidence intervals. * Adjusted *p* < 0.05; ** Adjusted *p* < 0.01; *** Adjusted *p* < 0.001.

β-catenin expression patterns demonstrated marked elevations in benign lesions with CEC, with notable shifts in subcellular localization. In endometrial polyps with CEC, total β-catenin expression (combining membranous, cytoplasmic, and nuclear positivity) showed 7.2-fold increase compared with polyps without CEC (mean: 43.2% vs. 6.0%; adjusted *p* < 0.001; d = 2.18). In polyps with atypical CEC, expression increased 9.8-fold (mean: 58.8% vs. 6.0%; adjusted *p* < 0.001; d = 2.87). Nuclear β-catenin localization was observed in 72% (13/18) of polyps with CEC compared with only 8% (2/25) of polyps without CEC (*p* < 0.001). As stated in the Introduction and revisited in Section 4.3, this nuclear β-catenin reactivity should not be interpreted as direct evidence of canonical Wnt-pathway activation in the absence of orthogonal molecular evidence. Comparative expression patterns across diagnostic categories are shown in Figure 2.

Hyperplasia with cystic/disordered glands showed similar patterns, with 9.1-fold increase in total β-catenin expression in cases with CEC (mean: 45.5% vs. 5.0%; adjusted *p* < 0.001) and nuclear localization in 65% of cases with CEC versus 5% of cases without. Hyperplasia with crowded glands demonstrated 7.2-fold increase (mean: 36.0% vs. 5.0%; adjusted *p* < 0.001) with nuclear positivity in 58% of lesions with CEC.

In established carcinomas, the pattern reversed. Endometrioid carcinoma with CEC showed 3.8-fold reduction in β-catenin expression compared with endometrioid carcinoma without CEC (mean: 16.9% vs. 64.3%; adjusted *p* < 0.001), with nuclear localization present in only 15% of cases with CEC versus 45% of cases without. Serous carcinoma showed similar reversal with 4.2-fold reduction in cases harboring ciliated change (mean: 13.5% vs. 56.9%; adjusted *p* < 0.001).

### 3.5. p53 Expression Patterns

In benign lesions (polyps and hyperplasia without atypia), p53 immunoreactivity showed a wild-type pattern irrespective of the presence of CEC, with no significant differences between areas with and without ciliated epithelium (all adjusted *p* > 0.3). These findings do not support p53 alteration as an early or consistent feature of benign CEC.

In endometrioid carcinoma, cases with CEC initially appeared to show higher p53 positivity on percentage-based review. After blinded re-review using pattern-based interpretive criteria to distinguish true aberrant overexpression from enhanced wild-type staining in metaplastic epithelium [26], this apparent difference was attenuated and p53 did not remain a robust discriminator of CEC in endometrioid carcinoma. These observations support cautious interpretation of p53 immunoreactivity in metaplastic or differentiation-associated endometrial epithelium.

Serous carcinoma showed the expected predominance of abnormal p53 staining overall. However, the subgroup of serous carcinomas containing CEC remained small, and the p53 findings are best regarded as exploratory. Taken together, the carcinoma-associated p53 observations do not support p53 as a primary marker of CEC and require molecularly integrated validation.

### 3.6. CD44 and Vimentin Expression

CD44 expression demonstrated lesion-dependent heterogeneity without a uniform direction of change. In endometrial polyps, CD44 was lower in areas with CEC, whereas some hyperplastic lesions showed higher expression in the ciliated component. Serous carcinoma with CEC also showed higher CD44 expression than serous carcinoma without such change. Given the inconsistency across categories, CD44 should be regarded as a secondary and exploratory marker in this dataset rather than a core finding.

Vimentin showed the most consistent pattern in the study, with reduced expression across all lesion types harboring CEC compared with their non-ciliated counterparts (all adjusted *p* < 0.05). Mean vimentin expression in ciliated epithelium ranged from 8.3% to 23.7%, versus 35.4% to 52.1% in corresponding non-ciliated epithelium. This reproducible reduction supports the interpretation that CEC is associated with a distinct differentiation-related phenotype rather than a conventional epithelial–mesenchymal transition profile. Complete marker-level data are provided in Supplementary Table S1.

### 3.7. Hormone Receptor and Proliferation Markers

Estrogen receptor and progesterone receptor expression varied across diagnostic categories without a consistent association with CEC. Ki-67 showed modest category-specific increases, most evident in atypical CEC within hyperplastic lesions. BCL2 and cyclin D1 displayed heterogeneous patterns and were not among the principal discriminatory markers after correction for multiple comparisons.

## 4. Discussion

**Figure 3:**
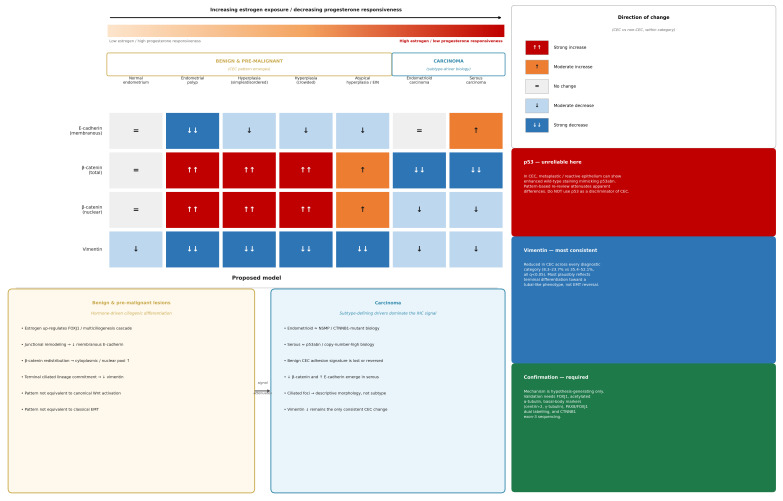
Schematic summary of key findings and proposed model. Top axis: increasing estrogen exposure/decreasing progesterone responsiveness. Horizontal axis: seven diagnostic categories (normal endometrium → endometrial polyp → simple/disordered hyperplasia → crowded hyperplasia → atypical hyperplasia/EIN → endometrioid carcinoma → serous carcinoma). Heatmap rows show the direction of change in CEC vs. non-CEC sub-cohorts for E-cadherin (membranous), β-catenin (total and nuclear), and vimentin (↑/↓/=). The proposed model panel summarizes that, in benign lesions, hormone-driven ciliogenic differentiation produces concurrent E-cadherin reduction, β-catenin redistribution and vimentin loss; in carcinoma the signal attenuates as molecular-subtype-defining drivers (NSMP/CTNNB1 and p53abn) dominate. Marginal annotations note that p53 is unreliable in this context, vimentin is the most consistent marker, and FOXJ1/CTNNB1 testing is required to confirm the proposed mechanism.

This exploratory study provides a systematic immunohistochemical survey of CEC across a broad spectrum of endometrial pathology using standardized digital quantification. Three points are most relevant for interpretation. First, benign lesions with CEC showed coordinated reduction in E-cadherin and increase in β-catenin, often with nuclear localization. Second, these patterns were not reproduced in carcinomas, indicating that ciliated differentiation in malignant lesions should not be interpreted as a simple extension of the benign pattern. Third, vimentin reduction was the most consistent finding across all categories, supporting a reproducible phenotype linked to ciliated differentiation. These data are best interpreted as comparative pathology observations that generate biologically plausible hypotheses but do not establish mechanism (Figure 3).

### 4.1. CEC in the Broader Context of Endometrial Differentiation, Hormonal Signaling and Endometriosis-Related Biology

Ciliated cells are an intrinsic, hormonally regulated component of the eutopic endometrial epithelium. Single-cell transcriptomic atlases of the cycling human endometrium consistently identify a discrete FOXJ1-expressing ciliated population that expands during the proliferative phase under estrogenic drive and contracts as progesterone dominance emerges in the secretory phase [5,6,7]. In endometrial organoid systems, sustained estradiol exposure together with NOTCH inhibition robustly induces multiciliogenesis (acetylated α-tubulin^+^/FOXJ1^+^), whereas WNT-pathway inhibition diverts differentiation toward the secretory lineage [6,30,31]. Against this background, the morphological observation termed in this manuscript ‘ciliated epithelial change’ cannot be interpreted as a single biological entity. It rather encompasses a continuum that ranges from physiologic, estrogen-driven multiciliation of normal proliferative or polypoid mucosa, through tubal-type metaplasia (true reprogramming toward fallopian-tube-like differentiation, characterized by an admixture of ciliated, secretory and intercalated cells, BCL2/PAX2 positivity, low Ki-67 and wild-type p53) [8,9,10,32], to the rare but biologically distinct ciliated-cell variant of endometrioid carcinoma [2,11]. Our findings should therefore be read as biomarker patterns observed across this continuum rather than as evidence of a uniform underlying process.

Ciliated differentiation also intersects with the pathobiology of endometriosis. Although our cohort comprises eutopic endometrial lesions and does not include endometriotic specimens, recent single-cell and spatial transcriptomic profiling of eutopic and ectopic endometrium has demonstrated that ciliated-cell proportions and FOXJ1/TPPP3 expression are altered in women with endometriosis: ovarian endometriomas display a bimodal distribution that includes a ‘fallopian-tube-like’ subtype with high FOXJ1^+^/PAX8^+^ ciliated content, whereas peritoneal and deep-infiltrating lesions retain a low ciliated frequency comparable to eutopic endometrium [33,34]. The eutopic endometrium of women with endometriosis is enriched in ciliated cells in the proliferative phase, suggesting that altered ciliogenic differentiation is a feature of the disease microenvironment rather than an exclusive property of ectopic implants [34]. The observation that CEC emerges in our cohort within conditions characterized by relative estrogen excess and progesterone unresponsiveness—endometrial polyps, hyperplasias, and to a lesser extent endometrioid carcinoma—invites parallels with the estrogen-dominance/progesterone-resistance paradigm that dominates current models of endometriosis pathogenesis [35].

Mechanistically, estradiol potently up-regulates the multiciliogenesis transcriptional cascade in endometrial epithelial cells, while progesterone restrains it indirectly via PR-mediated antagonism of estrogen-driven gene programs and through Wnt-pathway suppression [6,30]. In settings of unopposed estrogen action—anovulation, obesity-related peripheral aromatization, polyps, simple/disordered hyperplasia, tamoxifen exposure—the ciliated lineage expands, generating the morphologic appearance termed CEC [10,32]. We therefore interpret the immunohistochemical alterations associated with CEC in benign lesions (reduced E-cadherin, increased and partly nuclear β-catenin, reduced vimentin) as candidate hormone-context-dependent differentiation signatures rather than as autonomous pathobiological events. This conceptual framing is hypothesis-generating; formal confirmation will require co-staining with ciliogenic markers (FOXJ1, acetylated α-tubulin) and with molecular hormonal-state markers (PR-A/B, AR, FOXO1) in future work.

### 4.2. E-Cadherin/β-Catenin Alterations in Morphologic Context

The combination of lower E-cadherin expression and higher β-catenin expression in benign lesions with CEC is compatible with altered epithelial adhesion and redistribution of junction-associated proteins [36]. From a diagnostic perspective, this indicates that CEC may be accompanied by measurable immunophenotypic variation. However, immunohistochemistry alone cannot distinguish among differentiation-related change, altered protein turnover, context-dependent signaling, or specific molecular alterations. Accordingly, these findings should not be presented as proof of canonical Wnt-pathway activation or CTNNB1 alteration.

The carcinoma findings are equally important because they argue against a simple carryover of the benign pattern. Endometrioid and serous carcinomas containing CEC showed opposite or attenuated adhesion-marker patterns relative to benign lesions, implying that the meaning of ciliated differentiation depends on lesion context. In practice, CEC in carcinoma is best regarded as a descriptive morphologic phenotype rather than as a surrogate for the same biology observed in benign ciliated glands. For routine diagnostic work, the presence of ciliated cells should therefore prompt careful reassessment of the underlying architecture and cytology, but should not by itself move a lesion toward a benign, premalignant, or malignant interpretation.

### 4.3. β-Catenin in CEC: Explicit Caveats Against a Wnt-Pathway Interpretation

An important caveat is that nuclear β-catenin immunoreactivity, even when reproducibly observed, cannot be equated with canonical Wnt-pathway activation in the absence of orthogonal molecular evidence. β-catenin is a bifunctional protein whose membranous pool stabilizes cadherin-based adherens junctions while its nuclear pool acts as a TCF/LEF co-activator; the two pools are dynamically interconnected, and disruption of E-cadherin-mediated junctions, intracellular pH changes, post-translational modifications and steroid-hormone signaling can all redistribute β-catenin between compartments without invoking a CTNNB1 mutation [12,13]. In the endometrium specifically, estradiol enhances and progesterone restrains Wnt/β-catenin output during the menstrual cycle [32], and progesterone therapy itself can produce nuclear β-catenin accumulation in non-mutant tumors [37]. Even in carcinoma, the largest correlative study to date demonstrated that nuclear β-catenin staining is far from a perfect surrogate for CTNNB1 mutation, with substantial discordance in both directions [24,38]. We therefore explicitly caution against interpreting the nuclear β-catenin patterns reported here as evidence of canonical Wnt-pathway activation. Our findings are consistent with—but do not establish—Wnt-axis involvement, and equally with junctional remodeling, hormonally driven nucleocytoplasmic shuttling, or differentiation-state-specific β-catenin redistribution accompanying ciliated lineage commitment. Confirmation will require CTNNB1 exon-3 sequencing, validated downstream readouts (LEF1, AXIN2, cyclin D1 in TCF-bound contexts) and ideally co-localization with ciliogenic markers.

Published experimental work indicates that multiciliogenesis itself can involve stage-dependent shifts between canonical and non-canonical Wnt signaling [39], so the β-catenin patterns we observed remain compatible with a differentiation-coupled rather than oncogenic Wnt readout. This literature provides biologic context but should not be taken as direct support for a specific mechanism in the present cohort.

### 4.4. p53 Expression Patterns: Observations Requiring Molecular Validation

It is essential to recognize at the outset of this subsection that p53 immunohistochemistry, as currently interpreted in endometrial diagnostic practice, is calibrated to detect the TP53 mutation-associated patterns (mutant overexpression, null, cytoplasmic) that define the p53-abnormal molecular subgroup of endometrial carcinoma [18,26]. In benign and metaplastic endometrial epithelium—including CEC—p53 is therefore not a reliable marker of biological behavior and should not be expected to discriminate among the conditions studied here. In gynecologic pathology, metaplastic and reactive endometrial epithelium can show increased p53 staining without true TP53 abnormality, and pattern-based interpretation is essential [25,26,27]. After re-review, p53 did not provide strong discriminatory value in endometrioid carcinoma, and the unexpected serous carcinoma finding should be regarded as hypothesis-generating only. In the absence of sequencing and full molecular subclassification, p53 should not be used here as a central biologic argument.

### 4.5. Vimentin Downregulation: Implications for Differentiation

The uniform reduction in vimentin across all categories is the most reproducible finding in the manuscript. In descriptive terms, this suggests that CEC is associated with a relatively stable shift in epithelial phenotype. The observation does not by itself define lineage, mechanism, or epithelial–mesenchymal transition, but it appears more consistent than the heterogeneous changes seen in p53, CD44, BCL2, or cyclin D1.

Although the simplest reading of vimentin loss in epithelial neoplasia is reversal of an EMT-like program, this interpretation is unlikely to capture the biology of CEC for several reasons. First, normal endometrial epithelium constitutively co-expresses cytokeratin and a low level of vimentin throughout the menstrual cycle, and this baseline epithelial vimentin is itself dynamic, decreasing during ectopic and adenomyotic differentiation states [40] and during organoid-based ciliated-lineage commitment [31]. Second, fallopian-tube epithelium—the differentiation target of true tubal metaplasia—is comparatively vimentin-poor relative to glandular endometrial epithelium, so loss of vimentin in CEC is consistent with phenotypic convergence on a tubal-like terminally differentiated state rather than with mesenchymal transformation [5,6,41]. Third, single-cell endometrial atlases place ciliated cells at the terminal end of an epithelial differentiation trajectory whose late stages are characterized by down-regulation of mesenchymal-program transcripts and up-regulation of FOXJ1-driven ciliogenic genes [5,7]. We therefore propose that vimentin reduction in CEC reflects terminal differentiation along an alternative epithelial trajectory rather than mesenchymal-to-epithelial reversal of an oncogenic EMT. This reframing also accommodates the observation, made in independent endometrial-cancer cohorts, that retained or higher vimentin expression in carcinoma correlates with less aggressive behavior [42], and that ciliated-lineage markers (FOXJ1, p73, DYDC2, CTH) are positively associated with survival [41]. This hypothesis remains to be tested by direct co-localization of FOXJ1, acetylated α-tubulin and vimentin within single cells and by trajectory analysis of single-cell datasets stratified by CEC status.

The coexistence of reduced E-cadherin and reduced vimentin should not be overinterpreted through a simple epithelial–mesenchymal transition framework. In this setting, altered E-cadherin is more plausibly related to adhesion remodeling and β-catenin redistribution than to a full mesenchymal switch. The overall profile is therefore better described as context-dependent phenotypic remodeling than as classical EMT.

More broadly, immunohistochemistry is an informative but limited tool for interpreting metaplastic and differentiation-associated phenomena. Protein expression may vary according to hormonal state, maturation, degeneration, cellular stress, or post-translational regulation. Accordingly, the present results are best read as a carefully quantified immunophenotypic map, not as direct evidence of mutation, pathway activation, or clinical behavior.

### 4.6. Carcinoma Findings: Critical Examination of Alternative Hypotheses

Our finding that the immunohistochemical signature of CEC observed in benign lesions does not extend cleanly to endometrioid carcinoma—and is largely absent in serous carcinoma—is, in our view, the most biologically informative observation of this study and merits a more critical exploration than a conservative reading provides. At least four non-mutually exclusive interpretations require explicit consideration. (i) Degenerative or reactive phenotype. Ciliated cells in carcinoma may represent a reactive, non-neoplastic differentiation pattern induced by local inflammation, ulceration, or hormonal milieu, analogous to atypical tubal metaplasia, which despite mimicking serous intraepithelial carcinoma morphologically retains a benign immunoprofile (low Ki-67, wild-type p53, TERT-negative) and does not predict progression on long-term follow-up [9,43]. (ii) Treatment-induced phenotype. Progestin therapy in particular is known to provoke metaplastic differentiation—ciliated, mucinous, eosinophilic, secretory—within otherwise neoplastic glands and to induce nuclear β-catenin redistribution independent of CTNNB1 mutation [37]. We therefore re-examined our records and confirmed that none of the carcinoma cases studied had received pre-operative hormonal or radiation therapy, eliminating the most obvious version of this confounder, but cycle-phase effects and recent exogenous hormone exposure remain possible. (iii) Clonal heterogeneity within tumor. Endometrial carcinomas display substantial intratumoral genetic heterogeneity [44], and morphologically distinct foci within a single tumor can carry private mutations or sub-clonal copy-number changes. Without laser-microdissection-based comparison of CEC versus non-CEC components, we cannot determine whether the ciliated foci we scored are clonally derived from the surrounding carcinoma or represent reactive entrapped epithelium; recent demonstrations of rhizome-like clonal architecture in normal endometrium and persistence of mutant clones across morphologically variable glands [45] make both possibilities credible. (iv) Epiphenomenon unrelated to tumor biology. The pattern may simply reflect background CEC of the residual non-neoplastic endometrium being incidentally captured within carcinoma sections. The Hendrickson and Kempson [2] prototypic series of ciliated-cell carcinoma—and subsequent rare case reports [11]—establishes that bona fide ciliated neoplastic differentiation does occur, generally in low-grade endometrioid tumors with favorable behavior, consistent with the broader observation that retained ciliated-lineage marker expression (FOXJ1, p73) tracks with better endometrial-cancer survival [41]. We therefore propose, as the most parsimonious working model, that CEC in benign lesions and in low-grade endometrioid carcinoma reflects a hormone-permissive ciliated differentiation program superimposed on epithelium of varying clonal identity, while the loss of this signature in serous carcinoma reflects the TP53/copy-number-driven biology that dominates p53-abnormal tumors and is largely uncoupled from differentiation-dependent adhesion remodeling [14,18]. Discriminating among these hypotheses will require microdissection-based mutational comparison of CEC and non-CEC carcinoma compartments paired with FOXJ1/TAp73/acetylated-α-tubulin co-staining.

### 4.7. Clinical Significance of the 27.3% CEC Frequency

CEC was identified in 86 of 315 specimens (27.3%) in the present cohort. This frequency is broadly compatible with published estimates: in mixed-indication endometrial sampling series, ciliated/tubal metaplasia is reported as a frequent incidental finding (≈20–40%), with higher prevalence in proliferative/anovulatory endometrium, polyps, simple/disordered hyperplasia, and tamoxifen-exposed mucosa [10,43]. From a clinical standpoint, three implications follow. First, isolated CEC in the absence of cytologic atypia or hyperplastic architecture is not, in current evidence, an independent risk factor for subsequent malignancy: long-term follow-up studies demonstrate that even atypical tubal metaplasia carries a low progression rate similar to background endometrium [9]. Second, CEC is nevertheless a frequent diagnostic pitfall—it can mimic atypical hyperplasia, serous endometrial intraepithelial carcinoma and the rare ciliated-cell variant of endometrioid carcinoma [2,11]—and this is the principal reason its recognition matters clinically. Third, the appearance of CEC in obese, postmenopausal women with elevated estradiol levels has been associated with an immunoprofile that overlaps that of endometrial carcinoma in some cohorts, prompting the suggestion that CEC under specific clinical conditions may flag a hormonally permissive microenvironment for neoplastic progression [10]. The 27.3% frequency we observed therefore is best interpreted as expected for a hospital-based cohort enriched for hyperplastic and neoplastic lesions, and supports continued recognition of CEC as a diagnostically important but generally biologically benign morphological feature, whose context-dependent immunohistochemical alterations (the subject of this study) deserve further mechanistic study but should not currently alter clinical management.

### 4.8. Molecular Classification: Hypotheses Not Addressable by Immunohistochemistry Alone

Our study cannot be interpreted in isolation from the molecular classification framework that now defines endometrial carcinoma. Following TCGA [14], the ProMisE classifier [15,16] and its endorsement by the WHO 5th edition [4] and ESGO/ESTRO/ESP guidelines [17], endometrial carcinomas are stratified into four molecular subgroups—POLE-ultramutated, mismatch-repair-deficient (MMRd), p53-abnormal (p53abn, predominantly serous-like and copy-number-high), and no specific molecular profile (NSMP, copy-number-low)—each with distinct clinicopathologic features and outcomes [16]. CTNNB1 exon-3 hotspot mutations are essentially confined to the NSMP subgroup, where they are present in roughly 25–50% of cases and are independently associated with increased recurrence risk in early-stage low-grade endometrioid carcinoma [18,19,20]. By contrast, uterine serous carcinoma is biologically driven by TP53 alteration, copy-number instability and frequent HER2 amplification rather than by E-cadherin/adherens-junction or Wnt-axis pathobiology [18].

Several hypotheses raised by our descriptive findings cannot be evaluated within the present study design: (i) whether endometrioid carcinomas with CEC are enriched in CTNNB1-mutant or otherwise NSMP tumors, which would be biologically plausible given the established association of nuclear β-catenin with morular/squamous and rare ciliated differentiation patterns in NSMP [20,38]; (ii) whether the absence of distinctive E-cadherin/β-catenin/vimentin signatures in serous carcinomas with rare ciliated foci reflects the dominant TP53-driven, copy-number-high biology of this subgroup [18]; and (iii) whether the variable hormone-receptor and Ki-67 patterns we observed in CEC across categories track with the NSMP-ER^+^ versus NSMP-ER^−^ sub-stratification recently shown to refine NSMP prognosis [45]. These hypotheses can only be tested in a prospective cohort with concurrent molecular subtyping (POLE sequencing, MMR IHC, TP53/CTNNB1 sequencing, ± copy-number assessment) and we propose this as the most informative next step. Our IHC-only data should accordingly be regarded as exploratory and hypothesis-generating, intended to identify candidate phenotypes for molecularly resolved follow-up rather than to make claims about Wnt-axis or EMT pathway activation in defined molecular subgroups.

We therefore refrain from assigning prognostic significance to CEC. Although a differentiation-related interpretation is plausible, this study includes no outcome analysis, no direct ciliogenesis markers, and no integrated molecular classification. Any suggestion of favorable or unfavorable clinical behavior would therefore be premature and requires dedicated validation cohorts.

### 4.9. Study Limitations

Several limitations are important. First, this was an immunohistochemistry-only study without sequencing, transcriptomic analysis, direct assessment of ciliogenesis markers, or full molecular classification, so mechanistic interpretation remains limited. Second, although the cohort was broad, some subgroup analyses involved relatively small numbers of ciliated cases, especially within carcinoma subsets. Third, the study was retrospective and derived from a single institution. Fourth, clinical outcome data were not incorporated, precluding assessment of prognostic significance. Fifth, because ciliated cells can occur in normal endometrium, classification of CEC remains partly morphology-based despite strong inter-observer agreement.

A central methodological limitation deserving explicit emphasis is that we did not assess any direct molecular marker of multiciliogenesis. Mammalian motile ciliogenesis is governed by a defined transcriptional cascade in which GMNC (GEMC1) and MCIDAS, complexed with E2F4/5, activate downstream effectors including TAp73, MYB, CCNO, RFX2/3 and the master ciliogenic transcription factor FOXJ1, which in turn drives the structural axonemal program [39]. In endometrial epithelial organoids, this cascade is induced by estradiol and amplified by NOTCH inhibition [6,30]. None of these markers (FOXJ1, TAp73, GEMC1, MCIDAS, RFX2/3, CCNO) was evaluated in our cohort, nor were the canonical structural correlates of motile cilia—acetylated α-tubulin, polyglutamylated tubulin, IFT88/IFT20—or basal-body markers (centrin-2, γ-tubulin, pericentrin, SAS-6). Consequently, although our morphological diagnosis of CEC was rigorous, we cannot formally distinguish bona fide motile multiciliogenesis from morphological mimics, nor can we determine whether the immunohistochemical signatures reported here track with true ciliogenic-program activation. Future work from our group will incorporate a focused multiciliogenesis panel—minimally FOXJ1, acetylated α-tubulin and a basal-body marker (centrin-2 or γ-tubulin), with TAp73 and PAX8/FOXJ1 dual labeling to discriminate tubal-like (PAX8^+^/FOXJ1^+^) from physiologic endometrial (PAX8^−^/FOXJ1^+^) ciliated cells [34]—and will combine this with CTNNB1 sequencing to allow molecular-genetic anchoring of the morphological observations.

These limitations support a conservative, descriptive interpretation of the results. From a practical standpoint, the study is intended to reduce diagnostic overinterpretation rather than to promote marker-driven reclassification; morphology remains primary, and immunohistochemistry should be used only as contextual support when the differential diagnosis is otherwise established.

Strengths of the study include the comparatively large and systematically reviewed cohort, standardized digital quantification, explicit assessment of β-catenin localization, blinded ROI selection during image analysis, and the inclusion of a wide range of endometrial diagnostic categories. These features make the dataset valuable as a comparative pathology resource and as a foundation for future molecularly integrated studies.

## 5. Conclusions

This exploratory study shows that endometrial lesions with CEC have reproducible but context-dependent immunohistochemical alterations, most notably involving E-cadherin, β-catenin, and vimentin. In benign lesions, CEC is associated with reduced E-cadherin and increased β-catenin, frequently with nuclear localization; in carcinomas, these patterns are not maintained, arguing against a simple linear model across lesion categories. The most robust cross-category finding is vimentin downregulation, supporting a recurring phenotype associated with ciliated differentiation. We interpret this profile as most consistent with hormone-context-dependent terminal differentiation along the ciliated lineage, ranging from physiologic multiciliation to tubal-type metaplasia, rather than a Wnt-pathway- or EMT-driven oncogenic programme. Nuclear β-catenin reactivity in this setting should not be interpreted as evidence of canonical Wnt activation in the absence of CTNNB1 sequencing, and the carcinoma findings cannot be assigned to a defined TCGA/ProMisE molecular subgroup without integrated molecular data. The main practical message is that CEC can be encountered in benign, premalignant, and malignant endometrial lesions and should not be overinterpreted in isolation. Diagnostic classification should continue to rely primarily on the underlying glandular architecture and cytologic features, with immunohistochemistry serving as a contextual adjunct rather than a stand-alone discriminator of biologic behavior. These observations are descriptive and hypothesis-generating rather than mechanistic, and they should be validated in studies that integrate morphology with molecular classification, targeted sequencing, direct assessment of ciliogenesis-related markers (FOXJ1, TAp73, acetylated α-tubulin, basal body markers), and clinical outcomes.

## Figures and Tables

**Table 1:** Patient demographics and clinical characteristics by diagnostic category.

Diagnostic Category	*n*	Age, y (Mean ± SD)	Premenopausal *n* (%)	CEC *n* (%)	Atypical CEC *n* (%)
Normal endometrium	45	44.2 ± 7.8	28 (62.2)	8 (17.8)	3 (6.7)
Endometrial polyps	45	48.6 ± 8.2	18 (40.0)	15 (33.3)	7 (15.6)
Hyperplasia (cystic/disordered)	45	47.3 ± 9.1	16 (35.6)	17 (37.8)	7 (15.6)
Hyperplasia (crowded glands)	45	49.8 ± 8.7	12 (26.7)	14 (31.1)	7 (15.6)
Atypical hyperplasia/EIN	45	52.4 ± 9.3	8 (17.8)	12 (26.7)	7 (15.6)
Endometrioid carcinoma	45	57.1 ± 9.8	5 (11.1)	11 (24.4)	5 (11.1)
Serous carcinoma	45	62.8 ± 8.4	2 (4.4)	9 (20.0)	5 (11.1)
Total	315	51.7 ± 10.3	89 (28.3)	86 (27.3)	41 (13.0)

Abbreviations: SD, standard deviation; CEC, ciliated epithelial change.

**Table 2:** Mismatch repair protein status in carcinoma cases with and without ciliated epithelial change.

Histotype	CEC Status	MMR-Proficient *n* (%)	MMR-Deficient *n* (%)	MLH1/PMS2 Loss	MSH2/MSH6 Loss	Isolated Loss
Endometrioid	With CEC (*n* = 11)	5 (45.5)	6 (54.5)	4	2	0
Endometrioid	Without CEC (*n* = 34)	21 (61.8)	13 (38.2)	10	2	1
Serous	With CEC (*n* = 9)	7 (77.8)	2 (22.2)	0	2	0
Serous	Without CEC (*n* = 36)	34 (94.4)	2 (5.6)	0	0	2
Total	All (*n* = 90)	67 (74.4)	23 (25.6)	14	6	3

Abbreviations: MMR, mismatch repair; CEC, ciliated epithelial change. No significant association between MMR status and CEC status within histological subtypes.

**Table 3:** Selected immunohistochemical marker expression in lesions with CEC versus lesions without CEC.

Lesion Type	Marker	With CEC (Mean ± SD)	Without CEC (Mean ± SD)	Change	Adj. *p*
Endometrial polyps	E-cadherin	62.3 ± 12.4%	78.9 ± 8.7%	↓ 21.0%	0.003
Endometrial polyps	β-catenin	43.2 ± 18.6%	6.0 ± 3.2%	↑ 7.2×	<0.001
Endometrial polyps	Nuclear β-cat	72% positive	8% positive	↑ 9×	<0.001
Endometrial polyps	Vimentin	18.4 ± 9.2%	42.6 ± 14.3%	↓ 56.8%	<0.001
Hyperplasia (crowded)	E-cadherin	67.8 ± 11.2%	75.9 ± 9.8%	↓ 10.7%	0.021
Hyperplasia (crowded)	β-catenin	36.0 ± 15.8%	5.0 ± 2.8%	↑ 7.2×	<0.001
Hyperplasia (crowded)	Nuclear β-cat	58% positive	6% positive	↑ 9.7×	<0.001
Hyperplasia (crowded)	Vimentin	21.3 ± 10.1%	48.2 ± 15.6%	↓ 55.8%	<0.001
Atypical hyperplasia/EIN	E-cadherin	54.7 ± 14.8%	58.6 ± 12.4%	↓ 6.6%	0.041
Endometrioid carcinoma	β-catenin	16.9 ± 8.4%	64.3 ± 22.1%	↓ 3.8×	<0.001
Endometrioid carcinoma	Nuclear β-cat	15% positive	45% positive	↓ 3×	0.008
Serous carcinoma	E-cadherin	43.9 ± 16.2%	29.8 ± 11.4%	↑ 47.3%	<0.001
Serous carcinoma	β-catenin	13.5 ± 7.2%	56.9 ± 19.8%	↓ 4.2×	<0.001
Serous carcinoma	CD44	62.4 ± 21.3%	12.8 ± 6.4%	↑ 4.9×	<0.001

Abbreviations: Adj. *p*, adjusted *p*-value (Benjamini–Hochberg-corrected); Nuclear β-cat, nuclear β-catenin localization; CEC, ciliated epithelial change. ↑ indicates increase; ↓ indicates decrease.

## Data Availability

The raw data supporting the conclusions of this article will be made available by the corresponding author on reasonable request.

## Supplementary Materials

The following supporting information can be downloaded at: https://www.mdpi.com/article/10.3390/diagnostics16111646/s1, Table S1: Complete immunohistochemical marker expression data across the diagnostic categories stratified by the presence or absence of CEC; Table S2: Antibody catalog numbers and antigen-retrieval conditions; Supplementary Methods: Antibody details and digital pathology workflow.
