# Supplementary material for: Context-Dependent Alterations of E-Cadherin, β-Catenin, and Vimentin in Endometrial Ciliated Epithelial Change: An Exploratory Immunohistochemical Study
Source: Diagnostics (Basel). 2026 May 27;16(11):1646. doi: 10.3390/diagnostics16111646 (PMC13257306; doi:10.3390/diagnostics16111646)
Supplement: Supplementary file 1 [file diagnostics-16-01646-s001.zip › diagnostics-4242747-supplementary.pdf]

Supplementary Materials

Context-Dependent Alterations of E-Cadherin, β-Catenin, and Vimentin in Endometrial Ciliated Epithelial Change: An Exploratory Immunohistochemical Study

Teona Turashvili *, George Tevdorashvili, George Burkadze

Contents

Supplementary Table S1. Complete immunohistochemical marker expression data across all diagnostic categories
Supplementary Methods. Antibody details, staining protocol, and digital pathology workflow
Supplementary Table S2. Antibody catalog numbers and antigen-retrieval conditions

Supplementary Table S1. Complete Immunohistochemical Marker Expression Data Across All Diagnostic Categories Stratified by Ciliated Epithelial Change (CEC) Status

Diagnostic Category	Marker	No CEC (mean ± SD)	CEC (mean ± SD)	Atypical CEC (mean ± SD)	Change	Adj. p	Cohen's d
**Normal endometrium**	E-cadherin	76.2 ± 8.4%	71.4 ± 9.1%	68.9 ± 9.8%	**↓6.3%**	0.089	0.55
–	β-catenin (total)	4.8 ± 2.1%	8.2 ± 4.6%	10.1 ± 5.3%	**↑1.7×**	0.062	0.92
–	Nuclear β-catenin	0/37 (0%)	1/8 (12.5%)	0/3 (0%)	**–**	0.178	–
–	p53	3.2 ± 2.8%	4.1 ± 3.2%	4.4 ± 3.6%	**↑28.1%**	0.412	0.30
–	CD44	22.4 ± 9.3%	19.8 ± 8.7%	18.2 ± 7.9%	**↓11.6%**	0.341	0.29
–	Vimentin	46.3 ± 12.4%	28.6 ± 10.2%	24.3 ± 9.8%	**↓38.2%**	0.003	1.52
–	Estrogen receptor	82.1 ± 9.2%	79.4 ± 10.1%	77.8 ± 11.4%	**↓3.3%**	0.489	0.28
–	Progesterone receptor	78.4 ± 11.3%	74.2 ± 12.6%	71.6 ± 13.1%	**↓5.4%**	0.381	0.34
–	Ki-67	4.6 ± 3.2%	5.1 ± 3.6%	6.8 ± 4.2%	**↑10.9%**	0.623	0.14
–	BCL2	61.4 ± 14.8%	56.2 ± 13.9%	52.4 ± 15.2%	**↓8.5%**	0.291	0.36
**Endometrial polyps**	E-cadherin	78.9 ± 8.7%	62.3 ± 12.4%	58.9 ± 13.1%	**↓21.0%**	0.003	0.89
–	β-catenin (total)	6.0 ± 3.2%	43.2 ± 18.6%	58.8 ± 21.4%	**↑7.2×**	<0.001	2.18
–	Nuclear β-catenin	2/25 (8%)	13/18 (72%)	8/9 (89%)	**↑9×**	<0.001	–
–	p53	3.8 ± 2.9%	4.2 ± 3.4%	5.1 ± 4.1%	**↑10.5%**	0.538	0.13
–	CD44	31.2 ± 11.4%	22.6 ± 9.8%	19.4 ± 8.6%	**↓27.6%**	0.018	0.80
–	Vimentin	42.6 ± 14.3%	18.4 ± 9.2%	14.2 ± 7.8%	**↓56.8%**	<0.001	1.94
–	Estrogen receptor	74.3 ± 10.8%	69.8 ± 11.6%	66.2 ± 12.9%	**↓6.1%**	0.261	0.40
–	Progesterone receptor	68.9 ± 12.4%	62.4 ± 13.8%	58.7 ± 14.6%	**↓9.4%**	0.142	0.49
–	Ki-67	5.8 ± 4.1%	7.4 ± 4.8%	9.6 ± 5.3%	**↑27.6%**	0.214	0.36
–	BCL2	58.2 ± 15.3%	49.6 ± 14.2%	44.8 ± 13.9%	**↓14.8%**	0.098	0.58
**Hyperplasia (cystic/disordered glands)**	E-cadherin	74.6 ± 9.4%	62.8 ± 11.8%	59.3 ± 12.6%	**↓15.8%**	0.008	0.94
–	β-catenin (total)	5.0 ± 2.8%	45.5 ± 19.4%	61.2 ± 23.8%	**↑9.1×**	<0.001	2.31

Diagnostic Category	Marker	No CEC (mean ± SD)	CEC (mean ± SD)	Atypical CEC (mean ± SD)	Change	Adj. p	Cohen's d
–	Nuclear β-catenin	1/20 (5%)	11/17 (65%)	6/7 (86%)	**↑13×**	<0.001	–
–	p53	4.1 ± 3.1%	4.8 ± 3.7%	5.6 ± 4.2%	**↑17.1%**	0.481	0.20
–	CD44	28.4 ± 10.8%	24.1 ± 10.2%	21.6 ± 9.1%	**↓15.1%**	0.241	0.41
–	Vimentin	44.8 ± 13.7%	19.2 ± 10.4%	15.8 ± 8.9%	**↓57.1%**	<0.001	2.08
–	Estrogen receptor	76.8 ± 9.6%	72.4 ± 10.9%	68.9 ± 12.4%	**↓5.7%**	0.298	0.43
–	Progesterone receptor	71.2 ± 11.8%	65.6 ± 13.2%	61.4 ± 14.1%	**↓7.9%**	0.184	0.45
–	Ki-67	6.2 ± 4.4%	8.1 ± 5.2%	10.8 ± 5.9%	**↑30.6%**	0.182	0.39
–	BCL2	54.6 ± 14.6%	46.8 ± 13.4%	42.1 ± 12.8%	**↓14.3%**	0.119	0.56
**Hyperplasia (crowded glands)**	E-cadherin	75.9 ± 9.8%	67.8 ± 11.2%	63.4 ± 11.9%	**↓10.7%**	0.021	0.72
–	β-catenin (total)	5.0 ± 2.8%	36.0 ± 15.8%	48.4 ± 18.2%	**↑7.2×**	<0.001	2.14
–	Nuclear β-catenin	2/31 (6%)	8/14 (57%)	5/7 (71%)	**↑9.5×**	<0.001	–
–	p53	4.6 ± 3.4%	5.2 ± 3.9%	6.4 ± 4.8%	**↑13.0%**	0.561	0.16
–	CD44	34.8 ± 12.1%	28.4 ± 11.6%	24.8 ± 10.4%	**↓18.4%**	0.072	0.54
–	Vimentin	48.2 ± 15.6%	21.3 ± 10.1%	17.4 ± 9.2%	**↓55.8%**	<0.001	2.02
–	Estrogen receptor	72.4 ± 11.2%	66.8 ± 12.4%	62.4 ± 13.8%	**↓7.7%**	0.184	0.47
–	Progesterone receptor	66.8 ± 13.4%	59.4 ± 14.8%	54.6 ± 15.6%	**↓11.1%**	0.129	0.52
–	Ki-67	7.4 ± 4.8%	9.8 ± 5.6%	13.2 ± 6.4%	**↑32.4%**	0.148	0.45
–	BCL2	52.4 ± 14.8%	43.6 ± 13.2%	38.4 ± 12.6%	**↓16.8%**	0.086	0.62
**Atypical hyperplasia/EIN**	E-cadherin	69.4 ± 12.4%	58.9 ± 14.8%	54.7 ± 14.8%	**↓15.1%**	0.016	0.81
–	β-catenin (total)	18.4 ± 9.8%	28.6 ± 14.2%	36.4 ± 17.6%	**↑1.6×**	0.032	0.86
–	Nuclear β-catenin	5/33 (15%)	4/12 (33%)	3/7 (43%)	**↑2.2×**	0.164	–
–	p53	6.8 ± 4.6%	8.4 ± 5.2%	11.2 ± 6.4%	**↑23.5%**	0.312	0.33
–	CD44	42.6 ± 14.2%	38.4 ± 13.6%	34.8 ± 12.8%	**↓9.9%**	0.381	0.30
–	Vimentin	38.6 ± 14.4%	16.8 ± 9.4%	13.2 ± 8.1%	**↓56.5%**	<0.001	1.79
–	Estrogen receptor	64.8 ± 13.6%	59.2 ± 14.8%	54.6 ± 16.2%	**↓8.6%**	0.224	0.39
–	Progesterone receptor	58.4 ± 14.8%	52.1 ± 15.6%	47.8 ± 16.4%	**↓10.8%**	0.248	0.42
–	Ki-67	12.4 ± 6.8%	16.8 ± 7.6%	21.4 ± 8.2%	**↑35.5%**	0.076	0.60
–	BCL2	44.8 ± 14.2%	38.4 ± 13.6%	33.6 ± 12.8%	**↓14.3%**	0.149	0.46
**Endometrioid carcinoma**	E-cadherin	51.4 ± 18.6%	48.8 ± 19.2%	44.2 ± 18.8%	**↓5.1%**	0.684	0.14
–	β-catenin (total)	64.3 ± 22.1%	16.9 ± 8.4%	12.4 ± 6.8%	**↓3.8×**	<0.001	2.54
–	Nuclear β-catenin	15/34 (44%)	2/11 (18%)	1/5 (20%)	**↓2.4×**	0.008	–
–	p53	18.6 ± 12.4%	22.4 ± 14.8%	28.6 ± 16.2%	**↑20.4%**	0.389	0.28
–	CD44	48.4 ± 18.6%	41.2 ± 16.4%	38.6 ± 15.8%	**↓14.9%**	0.221	0.41
–	Vimentin	52.1 ± 18.4%	23.7 ± 11.2%	19.4 ± 9.8%	**↓54.5%**	<0.001	1.81
–	Estrogen receptor	52.4 ± 18.6%	48.6 ± 19.2%	44.8 ± 18.4%	**↓7.3%**	0.561	0.20
–	Progesterone receptor	44.6 ± 16.8%	40.2 ± 17.4%	36.8 ± 16.8%	**↓9.9%**	0.438	0.26
–	Ki-67	28.4 ± 14.8%	32.6 ± 16.2%	38.4 ± 17.6%	**↑14.8%**	0.341	0.27
–	BCL2	34.8 ± 14.6%	28.4 ± 12.8%	24.6 ± 11.4%	**↓18.4%**	0.148	0.46

Diagnostic Category	Marker	No CEC (mean ± SD)	CEC (mean ± SD)	Atypical CEC (mean ± SD)	Change	Adj. p	Cohen's d
**Serous carcinoma**	E-cadherin	29.8 ± 11.4%	43.9 ± 16.2%	46.2 ± 17.4%	↑**47.3%**	<0.001	1.34
–	β-catenin (total)	56.9 ± 19.8%	13.5 ± 7.2%	10.8 ± 5.6%	↓**4.2×**	<0.001	2.62
–	Nuclear β-catenin	6/36 (17%)	1/9 (11%)	0/5 (0%)	↓**1.5×**	0.481	–
–	p53	76.4 ± 22.8%	61.2 ± 24.6%	54.8 ± 26.2%	↓**19.9%**	0.088	0.64
–	CD44	12.8 ± 6.4%	62.4 ± 21.3%	68.4 ± 22.8%	↑**4.9×**	<0.001	2.91
–	Vimentin	35.4 ± 14.2%	8.3 ± 4.8%	6.4 ± 3.9%	↓**76.6%**	<0.001	2.34
–	Estrogen receptor	18.4 ± 9.6%	22.6 ± 11.4%	24.8 ± 12.6%	↑**22.8%**	0.261	0.40
–	Progesterone receptor	12.4 ± 7.8%	14.8 ± 9.2%	16.4 ± 10.4%	↑**19.4%**	0.391	0.28
–	Ki-67	62.4 ± 18.6%	58.6 ± 20.4%	54.2 ± 21.8%	↓**6.1%**	0.549	0.19
–	BCL2	18.4 ± 9.8%	14.2 ± 7.6%	12.4 ± 6.8%	↓**22.8%**	0.161	0.48

Abbreviations: CEC, ciliated epithelial change; Adj. p, adjusted P-value (Benjamini–Hochberg FDR correction); Cohen's d, effect size; –, not applicable or count-based comparison; ↑, increase; ↓, decrease; EIN, endometrial intraepithelial neoplasia; Nuclear β-catenin, proportion of cells with distinct nuclear staining ≥5%. Atypical CEC indicates cases with ciliated epithelial change accompanied by cytological atypia. All continuous data expressed as mean ± SD; nuclear β-catenin expressed as proportion of cases. Adjusted p-values from a total of 140 primary comparisons.

Note: Values for markers not specifically reported in the main text (ER, PR, Ki-67, BCL2) are derived from exploratory analysis and should be interpreted as supplementary and hypothesis-generating. Cyclin D1 was included in the immunohistochemical panel, is illustrated in Figure 1 of the revised manuscript, and is documented in Supplementary Table S2; its quantitative pattern was heterogeneous and non-discriminatory after correction for multiple comparisons.

Supplementary Methods. Antibody Details, Staining Protocol, and Digital Pathology Workflow

S1: Supplementary Table S2. Antibody Catalog Numbers and Antigen-Retrieval Conditions

All primary antibodies were commercially available and used according to manufacturer recommendations. Details of clone, manufacturer, catalog number, dilution, and antigen-retrieval conditions are summarized in Supplementary Table S2 and correspond to the antibody panel described in Section 2.6 of the revised main manuscript.

Antibody	Clone	Manufacturer	Catalog No.	Dilution	Antigen Retrieval	Expected Localization
E-cadherin	NCH-38	Dako (Agilent)	M3612	1:100	Citrate pH 6.0, 95°C, 20 min	Membrane
β-catenin	β-catenin-1	Dako (Agilent)	M3539	1:200	Citrate pH 6.0, 95°C, 20 min	Membrane / Cytoplasm / Nucleus
p53	DO-7	Dako (Agilent)	M7001	1:100	Citrate pH 6.0, 95°C, 20 min	Nucleus
CD44	DF1485	Dako (Agilent)	M7082	1:100	Citrate pH 6.0, 95°C, 20 min	Membrane
Vimentin	V9	Dako (Agilent)	M0725	1:200	Citrate pH 6.0, 95°C, 20 min	Cytoplasm
Estrogen receptor	1D5	Dako (Agilent)	M7047	1:100	Citrate pH 6.0, 95°C, 20 min	Nucleus
Progesterone receptor	PgR636	Dako (Agilent)	M3569	1:100	Citrate pH 6.0, 95°C, 20 min	Nucleus

Antibody	Clone	Manufacturer	Catalog No.	Dilution	Antigen Retrieval	Expected Localization
Ki-67	MIB-1	Dako (Agilent)	M7240	1:100	Citrate pH 6.0, 95°C, 20 min	Nucleus (proliferating cells)
BCL2	124	Dako (Agilent)	M0887	1:100	Citrate pH 6.0, 95°C, 20 min	Cytoplasm
Cyclin D1	SP4	Thermo Fisher	RM-9104	1:100	Citrate pH 6.0, 95°C, 20 min	Nucleus
MLH1	ES05	Dako (Agilent)	M3640	1:50	Citrate pH 6.0, 95°C, 20 min	Nucleus
PMS2	EP51	Dako (Agilent)	M3647	1:40	Citrate pH 6.0, 95°C, 20 min	Nucleus
MSH2	FE11	Dako (Agilent)	M3639	1:100	Citrate pH 6.0, 95°C, 20 min	Nucleus
MSH6	EP49	Dako (Agilent)	M3646	1:100	Citrate pH 6.0, 95°C, 20 min	Nucleus

S2. Automated Immunohistochemistry Platform

All immunohistochemical staining was performed on the Bond-Max automated immunostaining platform (Leica Biosystems, Wetzlar, Germany) using the Bond Polymer Refine Detection System (DS9800, Leica Biosystems). This polymer-based system employs a post-primary antibody and a compact polymer conjugated with HRP (horseradish peroxidase), providing enhanced sensitivity with low background.

Antigen retrieval: Heat-induced epitope retrieval (HIER) was performed using Bond Epitope Retrieval Solution 1 (citrate-based, pH 6.0) at 95°C for 20 minutes in all cases. Following retrieval, slides were allowed to cool in the buffer for 10 minutes prior to antibody incubation.

Chromogen: 3,3'-Diaminobenzidine (DAB) tetrachloride was applied for 10 minutes for chromogen development. Slides were counterstained with hematoxylin (Leica Bond Hematoxylin) for 5 minutes.

Controls: Each staining run included appropriate positive and negative controls. Positive controls consisted of endometrial carcinoma tissue sections for all markers, placental tissue for E-cadherin and β-catenin, and tonsil tissue sections for Ki-67 and p53. Negative controls were performed by omitting the primary antibody.

Batch control: All cases in each diagnostic category were processed in batches. Each batch included at least one positive and one negative control block. Inter-batch consistency was monitored by reviewing control slide staining intensity before scoring any case slides.

S3. Digital Pathology Workflow – Extended Detail

Whole slide imaging: All immunostained slides were digitized using an Aperio AT2 digital slide scanner (Leica Biosystems) at ×40 objective magnification (0.25 μm/pixel resolution). Slides were scanned in brightfield mode with automatic focus calibration. Scan quality was verified for each slide prior to analysis; slides with inadequate focus, significant air bubbles, or tissue folding were rescanned.

Software: Automated cell detection and quantification were performed using QuPath version 0.4.3 (open-source; https://qupath.github.io). Scripts were written in Groovy using the QuPath scripting interface for batch processing.

Cell detection: A cell detection algorithm was applied to each annotated region of interest (ROI). Cell detection parameters (nucleus radius range: 5–12 μm; minimum nucleus area: 30 μm^2; membrane detection: enabled) were optimized using an independent training set of 15 cases not included in the study cohort. Optical density thresholds were set using ROC analysis comparing automated scoring against blinded manual counting by two pathologists (inter-observer $\kappa = 0.84$ for automated vs. manual thresholds).

Staining classification: For each detected cell, cytoplasmic and nuclear DAB optical density (OD) values were computed. Cells were classified as positive or negative based on predetermined OD thresholds. For E-cadherin, membrane detection was additionally enabled to capture membrane-specific positivity. For β-catenin, separate quantification was performed for membrane, cytoplasmic, and nuclear compartments by adapting the subcellular localization classifier in QuPath.

ROI annotation: Region-of-interest (ROI) selection for digital quantification was performed by a third investigator who was blinded to CEC status. ROIs were selected to include representative epithelial areas and to exclude stroma, blood vessels, inflammatory infiltrate, necrosis, tissue folds, and poorly preserved regions. In cases with CEC, paired ROIs were annotated within the same section to capture areas with and without ciliated change at matched magnification. The median number of ROIs analyzed per case was 6 (interquartile range 4–8), and a minimum of 1000 epithelial cells was counted per ROI where tissue adequacy permitted.

Quality control: All automated counts were subject to visual quality control review. Cases where automated detection showed cell segmentation errors (fused nuclei, counted stroma) were manually corrected by adjusting ROI boundaries. Final quantitative data were exported as summary statistics per ROI and merged with case-level clinical data using R version 4.3.0.

S4. Statistical Analysis – Additional Details

Software: Statistical analyses were performed in SPSS Statistics version 29.0 (IBM Corp., Armonk, NY, USA) and R version 4.3.0 (R Foundation for Statistical Computing, Vienna, Austria). R packages used included ggplot2 (version 3.4.2) for visualization, rstatix (version 0.7.2) for group comparisons, and p.adjust() for multiple comparison correction.

Multiple comparison correction: The Benjamini–Hochberg (BH) procedure was applied to control the false discovery rate (FDR) at $\alpha = 0.05$ across 140 primary comparisons (10 markers × 7 diagnostic categories × 2 comparison types). Adjusted p-values (q-values) are reported throughout the main manuscript and Supplementary Table S1.

Effect size: Cohen's d was calculated for all continuous marker comparisons using pooled standard deviations. Values of $d \geq 0.2$, 0.5, and 0.8 were interpreted as small, medium, and large effects, respectively, following convention. Effect sizes were not calculated for count-based (proportional) comparisons; these are presented as ratios.

Normality and outlier assessment: Shapiro–Wilk tests were performed on all continuous variables within each subgroup. Variables failing normality ($p < 0.05$) were compared using Mann–Whitney U tests. Outliers were identified by the IQR method (>1.5 × IQR above the third quartile or below the first quartile) and verified by visual inspection of box plots. No cases were excluded solely on the basis of outlier status; exclusions were only made for tissue quality reasons.

End of Supplementary Materials
